# When teeth and kidneys fail together: a case series of amelogenesis imperfecta-renal syndromes in childhood

**DOI:** 10.11604/pamj.2025.52.51.48681

**Published:** 2025-10-01

**Authors:** Selsabil Nouir, Raja Boukadida, Safa Hannachi, Azza Harbi, Hajer Mokni, Houda Ajmi, Sameh Mabrouk

**Affiliations:** 1 Department of Pediatrics, Faculty of Medicine of Sousse, Sahloul University Hospital, University of Sousse, Sousse, Tunisia; 2 Department of Nephrology, Faculty of Medicine of Sousse, Sahloul University Hospital, University of Sousse, Sousse, Tunisia

**Keywords:** Amelogenesis imperfecta, dental enamel, tooth, kidney, renal insufficiency, renal tubular acidosis

## Abstract

Amelogenesis imperfecta (AI) is a group of hereditary conditions that affect enamel formation and may signal underlying systemic diseases. Although rare, renal involvement is increasingly being reported, particularly in the context of enamel-renal syndrome (ERS). This study describes and analyzes five pediatric cases of amelogenesis imperfecta associated with renal anomalies, aiming to raise awareness of this rare but significant syndromic association. We retrospectively examined the medical records of five children diagnosed with AI and renal pathology. A review of the clinical, biochemical, radiological, and dental findings was conducted. Outcomes and management strategies were analyzed. All patients exhibited enamel defects that were compatible with AI, ranging from hypoplasia to delayed eruption. The renal presentations were as follows: nephrocalcinosis, Bartter syndrome, chronic kidney disease, distal renal tubular acidosis, and ectopic calcifications. Two siblings presented with chronic kidney disease and tubular defects of unknown origin, raising suspicion of a hereditary tubulopathy. Despite the absence of molecular confirmation, the clinical profiles were suggestive of syndromic forms, such as Enamel Renal Syndrome (ERS) and WDR72-related conditions. AI should prompt a systemic evaluation, particularly renal screening. Pediatric dentists play a pivotal role in early detection. Multidisciplinary management, including nephrology and genetics, is essential for timely diagnosis and prevention of renal complications.

## Introduction

Dental enamel is the hardest and most mineral-rich tissue in the human body, composed of approximately 96% inorganic content, with the remainder consisting of water and organic matter. Disruptions in enamel formation can result from intricate interactions between genetic predispositions and environmental influences. Amelogenesis imperfecta (AI) refers to a diverse set of inherited disorders that impair the quantity and/or quality of enamel, and in some instances, may extend to affect other oral and systemic structures [1]. Its estimated prevalence ranges from 1 in 700 to 1 in 14,000 individuals, depending on the population studied [2]. AI can follow various patterns of inheritance, including autosomal dominant, autosomal recessive, X-linked, or appear sporadically. The sporadic presentations may result from de novo mutations, recessive transmission, or incomplete penetrance and variable expression of a dominant gene. The condition often displays a broad spectrum of clinical variability, even within the same family, and may affect primary, permanent, or both sets of dentition [3].

Multiple classification models have been introduced over the years [4]. The most widely recognized is Witkop´s system, which categorizes AI based on enamel characteristics into four main types: hypoplastic, hypocalcified, hypomaturation, and a mixed hypomaturation-hypoplastic form with taurodontism. When considering inheritance patterns alongside enamel phenotype, more than 15 subtypes have been described [5]. Although AI frequently occurs as an isolated dental anomaly, it can also be part of broader syndromic presentations. It has been observed in association with conditions such as cone-rod dystrophy, skeletal dysplasias like platyspondyly, pituitary dysfunction, and Kölschütter syndrome, among others [1,6].

Renal involvement, though uncommon, is gaining recognition in connection with AI. A specific condition known as enamel renal syndrome (ERS), characterized by the coexistence of AI and nephrocalcinosis, was first identified by MacGibbon in 1972 [7]. Other sporadic associations have been made between AI and renal disorders such as Bartter syndrome [3,8].

This study presents five pediatric cases diagnosed with AI and various forms of kidney disease, all managed in our pediatric nephrology department. Through this case series, we aim to provide insights into the clinical manifestations, biochemical profiles, therapeutic approaches, and renal prognoses, thereby enhancing current understanding of the link between AI and renal pathologies.

## Methods

**Study design and settings:** it is about a retrospective descriptive case series. The study was conducted at the Department of Pediatric Nephrology, Sahloul University Hospital, Sousse, Tunisia. Data were collected from patient records covering the period from January 2010 to December 2024.

**Participants:** we included five pediatric patients diagnosed with both AI and renal abnormalities. All were referred to the pediatric nephrology unit for renal complaints, with subsequent identification of associated dental anomalies. Inclusion criteria were the coexistence of confirmed AI and renal involvement. No exclusion criteria were applied.

**Variables:** key variables included demographic characteristics, family history (with a focus on consanguinity and kidney disease), clinical presentation (renal and dental), laboratory results (renal function, electrolyte balance, tubular markers), imaging findings, therapeutic interventions, and outcomes.

**Data sources and measurement:** data were retrieved retrospectively from paper medical records. All patients underwent thorough medical history reviews, including familial and perinatal backgrounds. Clinical examination was performed on admission. Laboratory tests included blood biochemistry (creatinine, electrolytes, acid-base status, PTH, vitamin D) and urine analysis (proteinuria, calcium, sodium, magnesium, phosphate handling). Renal imaging included ultrasonography in all cases; computed tomography (CT) scans were conducted when nephrocalcinosis or nephrolithiasis was suspected. Dental assessment was performed by a paediatric dentist through intraoral examination to assess enamel quality, tooth eruption, and gingival anomalies. Ophthalmological and metabolic evaluations were conducted as indicated.

**Bias:** selection bias was minimized by including all eligible cases within the study period. Information bias was limited by the use of standardized clinical and diagnostic protocols.

**Study size:** this case series included all identified patients meeting inclusion criteria within a 14-year period (n=5), reflecting the rarity of the condition.

**Quantitative variables:** quantitative variables such as serum creatinine, estimated glomerular filtration rate (eGFR), urinary electrolyte levels, and proteinuria, were recorded as absolute values and interpreted according to pediatric reference ranges.

**Statistical methods:** given the small sample size and descriptive nature of the study, no statistical comparisons or inferential analyses were performed. Data were summarized narratively and presented in tabular form.

## Results

The clinical data from our five cases are detailed in Table 1.

**Table 1:** summary of clinical, biological, and imaging findings in five patients with amelogenesis imperfecta and renal abnormalities

Case	Age / sex	Family history	Dental findings	Renal findings	Biochemical profile	Urinary abnormalities	Imaging findings	Diagnosis	Treatment & outcome
1	12 y/F	Consanguinity (2nd degree); grandfather with nephrolithiasis	Enamel hypoplasia	Stage 2 nephrocalcinosis; bilateral microcalculi	Normal blood tests; ↑ urinary Mg, ↓ urinary Ca	No proteinuria or hematuria	US: bilateral microcalculi (3-7 mm); CT: multiple non-obstructive stones	Enamel renal syndrome	Dental care; hydration; urine alkalinization; recurrent UTIs and renal colic; stable renal function
2	7 y/F	Consanguinity; two neonatal deaths in siblings	Generalized enamel defects; AI; retinitis pigmentosa	ESRD; nephromegaly; no stones	Hypokalemia, hyponatremia, alkalosis, ↑ renin and aldosterone, low Vit D, ↑ PTH	Proteinuria, microscopic hematuria, ↑ natriuresis, ↓ calciuria/magnesuria	US: nephromegaly; VCUG: bilateral VUR (Grade 2/3)	Bartter syndrome; chronic tubulointerstitial nephritis	Dialysis and renal transplant at age 8; favorable outcome post-transplant
3	12 y/F	Consanguinity; no relevant history	Retained deciduous teeth; hypoplastic yellow enamel; AI	Bilateral nephrolithiasis and nephrocalcinosis; ectopic calcifications	Normal renal function; slightly ↑ phosphate	Normal urinalysis; ↓ urinary calcium	US + CT: bilateral lithiasis; splenic, ovarian, and abdominal wall calcifications	AI with nephrocalcinosis and systemic calcifications	Dental care; hydration; recurrent renal colic; preserved renal function
4	17 y/M	Consanguinity; 3 deceased siblings with unknown etiology; 1 affected brother (case 5)	Thin, chipped enamel with structural loss; AI	CKD (eGFR 18); no lithiasis	Low Vit D; ↑ PTH; normal calcium/phosphate	Normal urinalysis	US: small kidneys; preserved corticomedullary differentiation	CKD of unknown origin with AI	Conservative management; dental care; stable CKD
5	14 y/M	Brother of case 4	Generalized yellow, rough enamel defects; AI	CKD with polyuria; pRTA disorder	Metabolic alkalosis, ↓ phosphate, ↑ Cl, ↑ PTH; normal Vit D	↑ urinary losses of Na, K, Mg; ↓ TRP	US: small kidneys, poor differentiation, right renal cyst	pRTA; CKD with AI	Electrolyte correction; bicarbonate supplementation; dental care; stable outcome

AI: amelogenesis imperfecta; US: ultrasonography; CT: computed tomography; VUR: vesicoureteral reflux; CKD: chronic kidney disease; ESRD: end-stage renal disease; PTH: parathyroid hormone; TRP: tubular reabsorption of phosphate; pRTA: proximal renl tubular acidosis; VCUG: voiding cystourethrography; VUR: vesicoureteral reflux

**Participants:** this case series includes five pediatric patients diagnosed with AI associated with renal abnormalities, all referred to the Pediatric Nephrology Department at Sahloul University Hospital between 2010 and 2024. All patients were born to consanguineous parents (mostly second-degree cousins). The patients were identified consecutively and represent all cases fulfilling the inclusion criteria within this 14-year period, with no exclusions, thereby minimizing selection bias.

**Figure 1:**
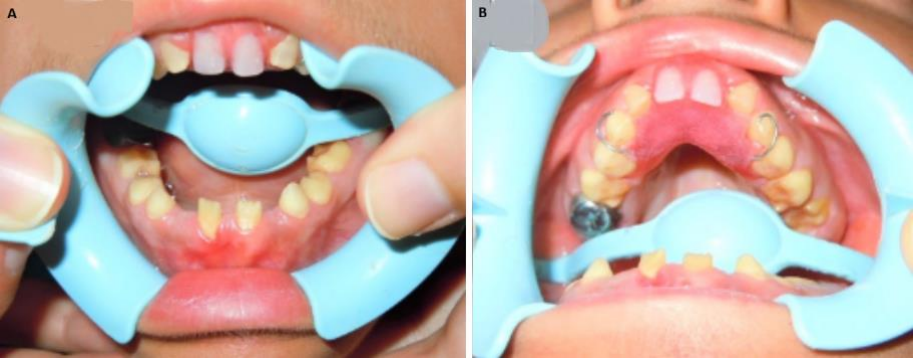
A,B) intraoral examination revealing hypoplastic, yellowish teeth presenting rough surfaces and conspicuous and irregular defects; the enamel alterations were generalized, excluding higher central incisors; the gingiva looked hypertrophied

**Descriptive data:** the median age at diagnosis was 12 years, with an age range from 7 to 15 years, indicating that the clinical manifestations of AI and associated renal pathology typically emerge in mid-to-late childhood. All patients exhibited classical clinical features of AI, including generalized enamel hypoplasia characterized by thin, rough, or absent enamel and frequently delayed eruption of permanent teeth (Figure 1). These dental features were strikingly uniform across cases and provided a vital diagnostic clue to an underlying systemic or syndromic disorder rather than isolated dental pathology.

A significant family history was documented in three of the five patients, including histories of renal disease, nephrolithiasis, or unexplained deaths of siblings during infancy or childhood (cases 4 and 5 had three deceased siblings from renal failure), reinforcing the suspicion of inherited renal involvement.

Most patients had normal anthropometric measures (height, weight, and BMI within age-appropriate percentiles) and normal developmental milestones. However, cases 4 and 5 presented with growth retardation, skeletal deformities with knee valgus and dysmorphic facial features, including mild midface hypoplasia and short stature, raising suspicion of a broader genetic syndrome affecting multiple organ systems.

### Outcome data

**Figure 2:**
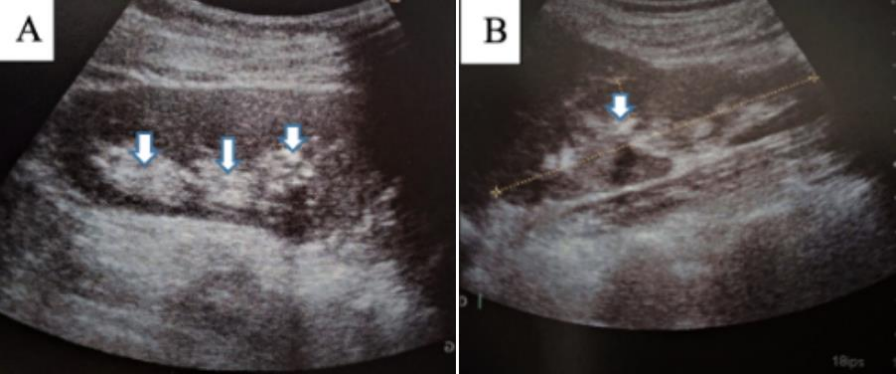
A,B) renal ultrasonography: mild medullar hyperechogenicity consistent with stage 2 nephrocalcinosis associated with multiple bilateral micro calculi

**Figure 3:**
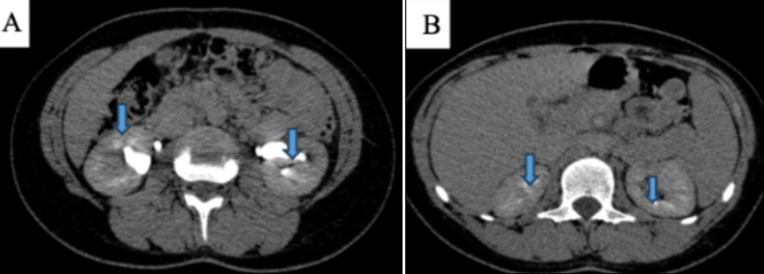
A,B) abdominal computed tomography confirming bilateral micro lithiasis (20 in the right kidney and 23 in the left side)

**Case 1:** presented with bilateral stage 2 nephrocalcinosis confirmed by ultrasonography and CT imaging (Figure 2, Figure 3). Laboratory evaluation revealed elevated urinary sodium and magnesium excretion, with hypocalciuria, indicating tubular electrolyte handling abnormalities. Despite recurrent urinary tract infections and renal colic during follow-up, renal function remained stable, with no progression to chronic kidney disease over several years. Dental management included restorative care, while nephrological management focused on hydration, urine alkalinization, and dietary sodium restriction.

**Case 2:** exhibited a complex clinical picture reminiscent of Bartter syndrome, including persistent hypokalemia, metabolic alkalosis, and salt wasting with elevated plasma renin and aldosterone levels. Renal imaging revealed bilateral nephromegaly without nephrocalcinosis or lithiasis. Urine studies showed markedly increased natriuresis and proteinuria alongside hypercalciuria and hypomagnesuria. Despite optimal medical therapy, she progressed rapidly to end-stage renal disease (ESRD) (eGFR 3 mL/min/1.73 m^2^) by age 8, necessitating renal transplantation. Ophthalmologic evaluation revealed retinitis pigmentosa, suggesting a syndromic multisystem disorder. Voiding cystourethrogram (VCUG) demonstrated bilateral vesicoureteral reflux (VUR) (grade 2 right, grade 3 left). Extensive metabolic and genetic testing excluded commonly known etiologies, suggesting a novel or rare genetic syndrome.

**Case 3:** presented with bilateral nephrolithiasis and extra-renal calcifications, including splenic, ovarian, and intraperitoneal deposits. Dental evaluation was consistent with AI (Figure 1). Laboratory investigations were largely normal except for hypocalciuria. The patient was managed conservatively with hydration, urine alkalinization, and a low-sodium diet. Although recurrent renal colic episodes occurred, kidney function remained preserved over time.

**Case 4:** presented with advanced chronic kidney disease (eGFR = 18 mL/min/1.73 m^2^) with stage 2 hypertension and was suspected to have tubulointerstitial nephritis based on clinical and imaging findings. Renal ultrasound demonstrated small kidneys with preserved corticomedullary differentiation but no nephrolithiasis or obstruction. Management included blood pressure control, treatment of secondary hyperparathyroidism, and dental care. He progressed to ESRD and is currently on hemodialysis.

**Case 5:** the younger sibling of case 4, presenting with enamel hypoplasia and skeletal deformities, including knee valgus. He had polyuria with a urine output of 2.5 L/m^2^/day but normal blood pressure and unremarkable urinalysis. Biochemical investigations revealed moderate renal impairment (eGFR 40 mL/min/1.73 m^2^), hypophosphatemia, metabolic alkalosis, hyperchloremia, and elevated parathyroid hormone, consistent with a proximal renal tubular acidosis (pRTA). Urine studies showed excessive losses of sodium, potassium, and magnesium, along with decreased tubular phosphate reabsorption. Renal ultrasound showed small kidneys with poor corticomedullary differentiation and a cyst in the right kidney. Treatment consisted of potassium and bicarbonate supplementation alongside supportive dental care.

**Main results:** across all five patients, there was clear clinical and radiological evidence linking AI with a heterogeneous spectrum of renal abnormalities, ranging from nephrocalcinosis and nephrolithiasis to chronic kidney disease and ESRD. Dental anomalies, particularly generalized enamel hypoplasia and delayed or abnormal tooth eruption, were consistent findings and served as an important clinical hallmark for systemic evaluation. Renal tubular dysfunction was a prominent feature, as evidenced by urinary abnormalities including elevated natriuresis, hypocalciuria, hypomagnesuria, and impaired tubular phosphate reabsorption. Imaging findings were diverse, showing nephrocalcinosis, nephrolithiasis, kidney size reduction, cystic lesions, and nephromegaly, suggesting variable renal pathologies within the same phenotypic spectrum.

**Other analyses:** due to the small sample size and observational design, formal statistical analyses or comparisons were not feasible. However, the clustering of cases in consanguineous families, the overlap of renal and dental phenotypes, and positive family histories strongly suggest a hereditary or syndromic condition linking enamel formation defects and renal tubular disease. Informed consent was obtained from all legal guardians of all participants prior to the commencement of the study. This was done in accordance with ethical research practices and applicable guidelines.

**Ethical approval:** the study was reviewed and approved by the ethics committee of Sahloul Hospital, which issued the required administrative and ethical clearance (Ethical Approval Number HS50-2025).

## Discussion

**Key results:** our five cases of AI with renal involvement broaden the clinical understanding of this condition and emphasize the critical need for comprehensive systemic assessment in affected children. Among our patients, patients 1 and 3 exhibited mild-to-moderate nephrocalcinosis with ectopic calcifications, consistent with classic FAM20A-related ERS. Dure-Molla *et al.* [9] emphasized that the pathognomonic oral profile includes generalized hypoplastic enamel, delayed eruption, gingival fibromatosis, and intrapulpal calcifications. Our patients presented several of these signs, strengthening the suspicion of underlying FAM20A mutations. Patient 2 presented with Bartter syndrome progressing to ESRD, alongside generalized AI and bilateral VUR, reinforcing rare associations between AI and salt-wasting tubulopathies [10]. Patients 4 and 5 were siblings from the same family with multiple early deaths related to unexplained renal disease. The older sibling (case 4) presented with chronic kidney disease of unknown etiology, associated with dental findings compatible with AI. His younger brother (case 5) presented with polyuria, yellowish hypoplastic enamel, hypophosphatemia, hyperchloremia, metabolic alkalosis, and small dysplastic kidneys with a renal cyst. The combination of tubular dysfunction and metabolic acidosis with hypophosphatemia raised the suspicion of pRTA. This clinical profile is consistent with recently described WDR72-related syndromes, where mutations in a vesicle trafficking gene lead to both enamel defects and tubular acidosis [11]. These clinical phenotypes align with previously described syndromic associations between AI and renal pathology [1,3,8,10,12]. Importantly, several of our patients displayed oral signs suggestive of specific genotypes, particularly FAM20A and WDR72 mutations, despite the lack of molecular confirmation due to local resource constraints. This case series provides additional evidence from North Africa, contributing to the growing body of literature recognizing the link between AI and systemic disorders, especially renal disease [9,10,11,13].

**Limitations:** this study has several limitations. First, molecular genetic testing was not available for any of the patients due to financial and technical constraints. This limits the ability to definitively confirm the suspected underlying genotypes, such as FAM20A or WDR72 mutations. Additionally, the small sample size and retrospective nature of the data collection restrict the generalizability and statistical power of our observations. In some families, follow-up data were incomplete, particularly regarding other potentially affected siblings who died prematurely. These factors introduce potential selection and reporting bias, and the full extent of renal involvement may be under- or overestimated.

**Interpretation:** clinically, AI exhibits significant variability. It may present as an isolated condition or occur in conjunction with various syndromic abnormalities, including cone-rod dystrophy, platyspondyly, hypothalamo-hypophyseal insufficiency, Kohlschütter syndrome, and tricho-dento-osseous (TDO) syndrome [14,15]. While these systemic associations are well-documented, reports linking AI specifically with renal pathology remain relatively scarce in the literature. Despite these limitations, our findings reinforce previous reports that AI is not merely a dental disorder but may be the earliest manifestation of broader systemic disease, particularly renal dysfunction. The clinical variability among our cases, ranging from isolated nephrocalcinosis to ESRD and tubular disorders, mirrors the genetic heterogeneity previously described in the literature [8, 10, 16].

The ERS, characterized by the co-occurrence of AI and nephrocalcinosis, was first described by MacGibbon in 1972 in a non-consanguineous sibling pair [7]. Subsequent reports have documented this association in both consanguineous and non-consanguineous families, typically following an autosomal recessive inheritance pattern [16,17]. Beyond nephrocalcinosis, other renal manifestations associated with AI include hypocalciuria, impaired urinary concentrating ability, Bartter syndrome, and renal tubular acidosis [1, 3, 18]. Amelogenesis imperfecta (AI) can follow various inheritance patterns, including autosomal dominant, autosomal recessive, X-linked, or appear as sporadic cases [19]. Among the recessive forms, mutations in the FAM20A gene are now well recognized as the cause of ERS, a condition defined by generalized hypoplastic enamel defects and early nephrocalcinosis, often with ectopic calcifications as described in case 3 [13]. Additionally, alterations in the WDR72 gene have been associated with AI occurring alongside renal tubular acidosis and nephrocalcinosis [11]. Other syndromic variants of AI, particularly those involving mutations in SLC12A1 or KCNJ1, may present clinical features similar to Bartter syndrome and can progress more rapidly toward ESRD [10]. Our findings also highlight the role of dental signs, such as generalized hypoplastic enamel, delayed eruption, and gingival fibromatosis, as early indicators of potential renal disease [9]. In the absence of genetic confirmation, clinical and radiological features remain essential for risk stratification and multidisciplinary referral. Furthermore, we highlight that some systemic features may only manifest later, underscoring the need for long-term monitoring even in apparently mild cases.

**Generalisability:** although our case series includes a small number of patients, it adds valuable insights from a North African population, where consanguinity is common and may contribute to the expression of autosomal recessive disorders such as FAM20A- and WDR72-related syndromes. These findings may be applicable to other regions with similar demographic and genetic characteristics. However, generalizing our results to broader populations must be done cautiously due to sample size and lack of genetic confirmation.

Nonetheless, our recommendations for systematic renal screening in all patients diagnosed with AI, multidisciplinary collaboration, and pursuit of genetic testing, when possible, are relevant across diverse clinical settings. They support a preventive approach that could improve outcomes in affected children, particularly when diagnosis occurs early and surveillance is ongoing.

## Conclusion

These clinical observations reveal that amelogenesis imperfecta (AI) frequently coexists with renal impairment, highlighting the need for systematic kidney function screening in all diagnosed cases. The window of opportunity for preventing renal complications depends crucially on timely identification, making dental professionals the frontline detectors of systemic involvement. Our experience confirms that standard care protocols for AI patients should incorporate detailed nephrological history-taking, baseline serum creatinine and electrolyte panels, and renal ultrasound imaging regardless of symptoms. This tripartite approach ensures no affected individual misses early intervention that could preserve renal function.

***What is known about this topic***

- *Amelogenesis imperfecta (AI) is a genetically heterogeneous condition affecting enamel development, and it may occur in isolation or as part of syndromes;*
- *Rare associations between AI and renal anomalies, such as nephrocalcinosis or Bartter syndrome, have been reported in the literature;*
- *Enamel-renal syndrome is a recognized but uncommon entity linking dental and kidney abnormalities through shared genetic pathways.*

***What this study adds***

- *This case series highlights the clinical variability of renal involvement in children with AI, including nephrocalcinosis, Bartter syndrome, distal renal tubular acidosis, and end-stage renal disease;*
- *It underscores the importance of systematic renal evaluation in children diagnosed with AI, especially in consanguineous families;*
- *The findings support a multidisciplinary approach involving pediatric nephrologists and dentists for early detection and better long-term outcomes.*
